# Tailing miniSOG: structural bases of the complex photophysics of a flavin-binding singlet oxygen photosensitizing protein

**DOI:** 10.1038/s41598-019-38955-3

**Published:** 2019-02-20

**Authors:** Joaquim Torra, Céline Lafaye, Luca Signor, Sylvain Aumonier, Cristina Flors, Xiaokun Shu, Santi Nonell, Guillaume Gotthard, Antoine Royant

**Affiliations:** 1 Institut Químic de Sarrià, Universitat Ramon Llull, Via Augusta 390, Barcelona, 08017 Spain; 2 Univ. Grenoble Alpes, CNRS, CEA, IBS (Institut de Biologie Structurale), F-38000 Grenoble, France; 3 European Synchrotron Radiation Facility, F-38043 Grenoble, France; 4 Madrid Institute for Advanced Studies in Nanoscience (IMDEA Nanoscience), Ciudad Universitaria de Cantoblanco, C/Faraday 9, 28049 Madrid, Spain; 5 Nanobiotechnology Unit Associated to the National Center for Biotechnology (CNB-CSIC-IMDEA), Ciudad Universitaria de Cantoblanco, 28049 Madrid, Spain; 6 Department of Pharmaceutical Chemistry, University of California-San Francisco, San Francisco, California 94158-9001 United States; 7 Cardiovascular Research Institute, University of California-San Francisco, San Francisco, California 94158-9001 United States

## Abstract

miniSOG is the first flavin-binding protein that has been developed with the specific aim of serving as a genetically-encodable light-induced source of singlet oxygen (^1^O_2_). We have determined its 1.17 Å resolution structure, which has allowed us to investigate its mechanism of photosensitization using an integrated approach combining spectroscopic and structural methods. Our results provide a structural framework to explain the ability of miniSOG to produce ^1^O_2_ as a competition between oxygen- and protein quenching of its triplet state. In addition, a third excited-state decay pathway has been identified that is pivotal for the performance of miniSOG as ^1^O_2_ photosensitizer, namely the photo-induced transformation of flavin mononucleotide (FMN) into lumichrome, which increases the accessibility of oxygen to the flavin FMN chromophore and makes protein quenching less favourable. The combination of the two effects explains the increase in the ^1^O_2_ quantum yield by one order of magnitude upon exposure to blue light. Besides, we have identified several surface electron-rich residues that are progressively photo-oxidized, further contributing to facilitate the production of ^1^O_2_. Our results help reconcile the apparent poor level of ^1^O_2_ generation by miniSOG and its excellent performance in correlative light and electron microscopy experiments.

## Introduction

miniSOG (for mini Singlet Oxygen Generator)^1^ is a 106 amino acid flavin-binding protein that generates ^1^O_2_ under exposure to blue light. It was originally developed by Shu and coworkers for correlative light and electron microscopy (CLEM) as it both fluoresces and catalyzes the photo-oxidation of diaminobenzidine (DAB), providing high-resolution images^1^. Novel applications are being actively developed since^2–5^. miniSOG was engineered from the LOV2 (Light, Oxygen and Voltage) domain of *Arabidopsis thaliana* phototropin 2^1^. Proteins based on LOV domains are blue-light photoreceptors that form a light-induced and reversible flavin-cysteine covalent adduct that consumes the energy of the excited state^6^. Replacement of the cysteine residue by an alanine or glycine avoids the formation of the covalent bond and leads to a fluorescent protein^7,8^. miniSOG contains six mutations as compared to its precursor, two of them involving residues surrounding the chromophore. Its cofactor FMN is ubiquitously found in nature^9,10^ and generates ^1^O_2_ with high quantum yield (*Φ*_∆_)^11^, but also other reactive oxygen species (ROS)^12^.

Close inspection of the photophysical and photosensitizing properties of miniSOG reveals a number of striking observations: (1) its *Φ*_∆_ is much lower than that of FMN (0.03 vs. 0.51)^11,13,14^; (2) the lifetime (τ_T_) of triplet miniSOG (^3^miniSOG*) is much shorter than that of FMN in nitrogen-saturated solutions (33.6 µs^15^ vs 200 µs^16^); (3) oxygen quenching is less efficient than for FMN (τ_T_^air^ = 31.3 µs^15^ vs 3.1 µs in air-saturated solutions); (4) in addition to ^1^O_2_ it also produces superoxide (O_2_^•^^−^)^12,14^; (5) it undergoes a remarkable transformation upon exposure to light, whereby *Φ*_∆_ increases 10-fold (to ~0.3) and τ_T_^air^ shortens by 10-fold (to ~3 µs)^13,14^. The absence of a structure of miniSOG so far had prevented to rationalize these observations, which we have attempted here using a combined structural and photophysical approach.

**Figure 1:**
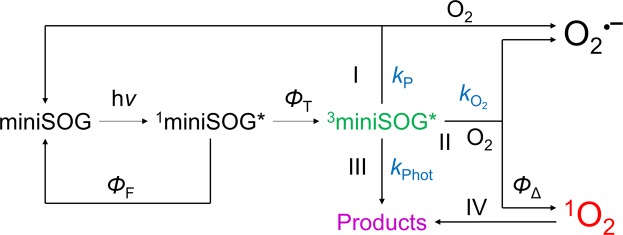
Model of oxygen photosensitization by miniSOG. *Φ*_F_: fluorescence quantum yield, *Φ*_T_: triplet state quantum yield, *k*_P_: protein quenching rate constant, *k*_Phot_: photoproduct formation rate constant, ${k}_{{{\rm{O}}}_{2}}$: oxygen quenching rate constant, *Φ*_Δ_: singlet oxygen quantum yield. I, II, III, and IV indicate the three deactivation and one oxidation pathways discussed in the main text.

Based on the extensive data present in the literature and the photophysical and structural results presented herein, a mechanism of excited-state deactivation of miniSOG can be proposed that involves three main pathways (Fig. 1). The shorter lifetime of ^3^miniSOG* compared to ^3^FMN* indicates that protein quenching is a major mechanism of triplet decay (pathway I). Its rate constant *k*_P_ is largely determined by electron transfer with nearby electron-rich residues^17^. Quenching of the singlet state can be safely ruled out since no shortening of the fluorescence lifetime or decrease in the fluorescence quantum yield are observed relative to free FMN. In the presence of oxygen, a second decay pathway (pathway II) is possible, namely oxygen quenching to produce ^1^O_2_ (energy transfer) or O_2_^•−^ (electron transfer), as observed for FMN in solution^12^. It is also possible to produce O_2_^•−^ by reaction of oxygen with a radical anion formed during protein quenching in pathway I. Finally, miniSOG undergoes a photoinduced transformation (pathway III, rate constant *k*_Phot_), for which we provide here a detailed description for the first time.

## Results and Discussion

### High resolution crystal structure of miniSOG

**Figure 2:**
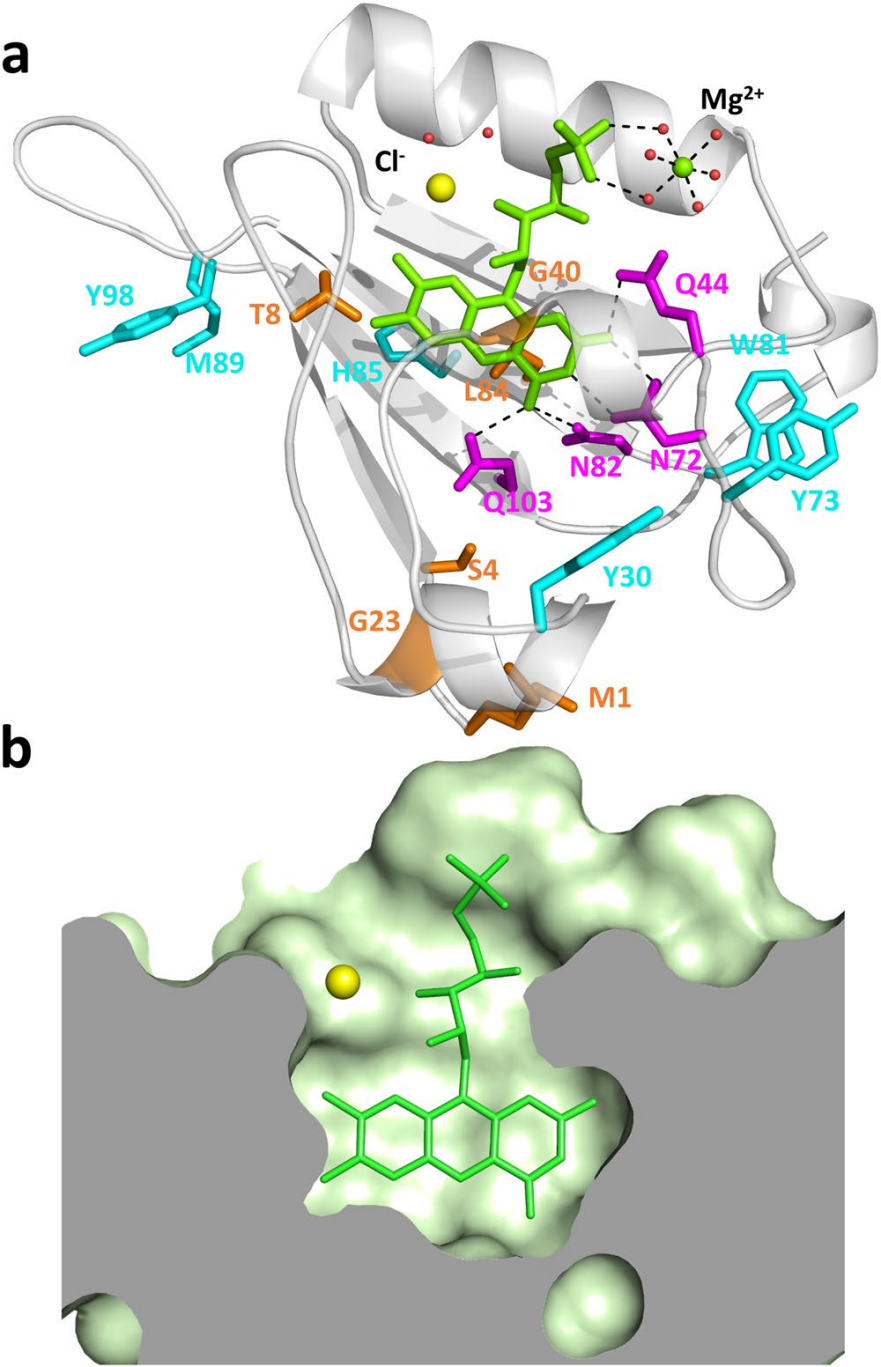
High-resolution crystallographic structure of miniSOG. (**a**) Secondary structure (white) represented with FMN (green), chloride (yellow), magnesium (green) and water molecules (red). Represented residues: mutations from LOV2 (orange), residues hydrogen-bonded to the FMN ring (magenta), and potential quenchers of ^3^FMN* (cyan). (**b**) Topology of the FMN-binding site.

We have solved the structure of miniSOG at 1.17 Å resolution (Fig. 2a and Supporting Information), which shows an increase in rigidity of the environment of the chromophore compared to that in the LOV2 domain, the location of potential quenchers of the excited states of FMN, and the phosphoribityl tail of FMN lying in a tunnel bridging the bulk solvent and the chromophore encased in the core of the protein (Fig. 2b). The latter hinders oxygen access to the isoalloxazine ring. The presence halfway through the tunnel of a chloride ion, which can be a good mimic of molecular oxygen^18,19^, suggests that oxygen diffusion can occur.

### Deactivation mechanism of miniSOG triplet excited state (Pathways I and II)

The values of the relevant rate constants for pathways I and II can be inferred from the ^3^miniSOG* lifetime measurements. Comparison of the decay rate constant (1/τ_T_) of miniSOG and SOPP3, the miniSOG mutant with the longest τ_T_ reported so far (3.3 ms in nitrogen-saturated solutions)^17^ allows us to estimate the rate constant for protein quenching (*k*_P_ = *k*_T_^N2^ − *k*_T_^N2^_,SOPP3_, Table 1). SOPP3 is a miniSOG variant, which encases the same chromophore FMN and, most importantly, lacks most of the electron-rich residues present in the vicinity of the flavin in miniSOG. Hence, protein quenching of the triplet chromophore in SOPP3 is essentially suppressed, which makes SOPP3 a convenient model for the study of the contribution of protein quenching in miniSOG. Likewise, the pseudo-first order rate constant for oxygen quenching (${k}_{{{\rm{O}}}_{2}}$ = *k*_T_^Air^ − *k*_T_^N^_2_) can be estimated from τ_T_ data in air- and nitrogen-saturated solutions (Table 1).

**Table 1:** Photophysical properties of miniSOG in D_2_O- and H_2_O-based phosphate buffer.

Parameter	in D_2_O	in H_2_O	Pathway	References
τ_S_	5.0 ns	4.9–5.5 ns		^13, 15, 43^
*Φ*_F_	0.43	0.37–0.44		^1, 14, 15, 43, 44^
*Φ*_T_	0.6	0.6		^15^
*Φ*_Δ_	0.03–0.04	0.03–0.05		^13, 14, 23, 31, 44^
${k}_{{\rm{T}}}^{{{\rm{N}}}_{2}}$	2.41 × 10^4^ s^−1^	2.98 × 10^4^ s^−1^		^15^
*k*_**P**_	**2.38** × **10**^**4**^ **s**^**−1**^	**2.95** × **10**^**4**^ **s**^**−1b**^	**I**	This work
*k*_T_^Air^	2.59 × 10^4^ s^−1^	3.19 × 10^4^ s^−1^		^15^
${{\boldsymbol{k}}}_{{{\bf{O}}}_{{\bf{2}}}}$	**1.8** × **10**^**3**^ **s**^**−1**^	**2.3** × **10**^**3**^ **s**^**−1**^	**II**	This work
*k*_**Phot**_	**6.0** **s**^**−1**^	—	**III**	This work

D_2_O was used to increase the singlet oxygen lifetime, thus boosting the reactions and processes in which singlet oxygen is involved and facilitating its detection^45^.

^a^Assuming the same value of *k*_T_^N2^_,SOPP3_ in H_2_O and D_2_O.

Comparison of *k*_p_ and ${k}_{{{\rm{O}}}_{2}}$ in Table 1 reveals that protein quenching (pathway I) is the main triplet deactivation pathway, removing 93% of the triplets in air-saturated solutions *k*_P_/(*k*_P_ + ${k}_{{{\rm{O}}}_{2}}$). Oxygen only quenches 7% of the triplets, which limits *Φ*_∆_ to 0.6 × 0.07 = 0.042 (Eq. 1), in excellent agreement with the experimental value. It can therefore be concluded that the modest *Φ*_∆_ of miniSOG is due to an unfavorable combination of low ${k}_{{{\rm{O}}}_{2}}$ and high *k*_P_ values, as proposed previously^17^.

1 $${{\rm{\Phi }}}_{{\rm{\Delta }}}={{\rm{\Phi }}}_{{\rm{T}}}\times \frac{{k}_{{{\rm{O}}}_{2}}}{{k}_{{\rm{p}}}+{k}_{{\rm{P}}{\rm{h}}{\rm{o}}{\rm{t}}}+{k}_{{{\rm{O}}}_{2}}}\approx {{\rm{\Phi }}}_{{\rm{T}}}\times \frac{{k}_{{{\rm{O}}}_{2}}}{{k}_{{\rm{p}}}+{k}_{{{\rm{O}}}_{2}}}$$

Our structural results above suggest that the low value of ${k}_{{{\rm{O}}}_{2}}$ is due to the steric hindrance of the ribityl tail within the tunnel which provides oxygen access to the FMN. Regarding *k*_P_, the miniSOG structure shows that six electron-rich residues are positioned within 8.2 to 10.2 Å from the isoalloxazine ring, namely Tyr30, Tyr73, Trp81, His85, Met89 and Tyr98, and are thus close enough to the chromophore to act as electron-transfer quenchers of ^3^miniSOG*^20^. In addition, four hydrophilic residues, Glu44, Asp72, Asp82 and Glu103, form hydrogen bonds with FMN, and may thus enhance protein quenching and O_2_^•−^ formation^21^. Replacing selectively these residues should lead to a lengthening of the triplet lifetime of miniSOG^22^ and hence to a higher fraction of triplets being trapped by oxygen, thus to a higher *Φ*_∆_ value. In fact, some of these positions have already been mutated in light of their capacity of direct electron transfer from the FMN: such miniSOG mutants show considerably longer τ_T_ values (e.g., 196 µs for miniSOG Q103L (SOPP)^23^, 1.1 ms for miniSOG W81F (Supplementary Fig. S2), and 3.3 ms for SOPP3^17^ in oxygen-free solutions) and larger *Φ*_∆_ values (0.25, 0.33 and 0.6, respectively), in agreement with Eq. 1. It is worth noting also that miniSOG produces more O_2_^•−^ than free FMN^12^, which indicates that the radical anion pathway contributes to the production of O_2_^•−^. Indeed, SOPP shows an 8-fold higher *Φ*_∆_ value than miniSOG but only a 1.3 higher yield of O_2_^•−^ ^23^. Thus, removal of hydrophilic side chains in the vicinity of the chromophore should strongly reduce the relative formation of O_2_^•−^ *vs*. ^1^O_2_.

### Consequences of blue-light irradiation of miniSOG on its FMN chromophore (Pathway III)

**Figure 3:**
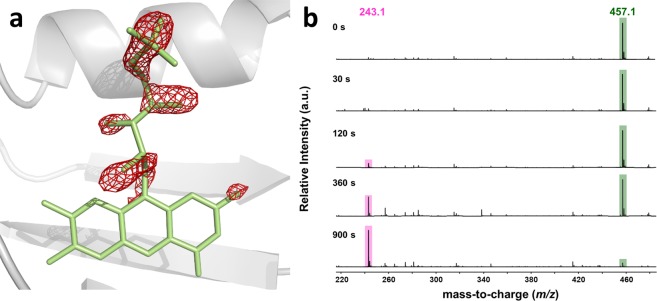
Blue-light induced structural changes on the chromophore of miniSOG. (**a**) 2.0 Å resolution difference Fourier map calculated between non-irradiated and irradiated parts of a miniSOG crystal contoured at a −3.0 σ level (magenta) superimposed on the FMN molecule (green). (**b)** ESI-TOF mass spectra acquired in the low mass range (*m*/z < 500) of miniSOG progressively irradiated with blue-light.

In light of Eq. 1, the observed 10-fold decrease in τ_T_ and similar increase in *Φ*_∆_ upon extended photolysis suggest severe changes in both *k*_P_ and ${k}_{{{\rm{O}}}_{2}}$. Blue-light (440 nm) irradiation of a miniSOG crystal at 10 W·cm^−2^ led to a five-fold decrease of the fluorescence signal over a 30 min course (Supplementary Fig. S3) and was gentle enough to keep diffraction around 2.0 Å resolution while affecting a sufficient fraction of molecules so that structural alterations could be visualized in electron density maps. A difference Fourier map calculated from non-irradiated and irradiated parts of a crystal revealed the loss of electron density all along the ribityl tail of the FMN (Fig. 3a), strongly suggesting its cleavage. Besides, Electrospray ionisation time-of-flight (ESI-TOF) mass spectrometry performed on irradiated protein samples show (Fig. 3b) the progressive disappearance of the FMN peak at *m*/*z* = 457.1 in favor of a peak at *m*/*z* = 243.1.

**Figure 4:**
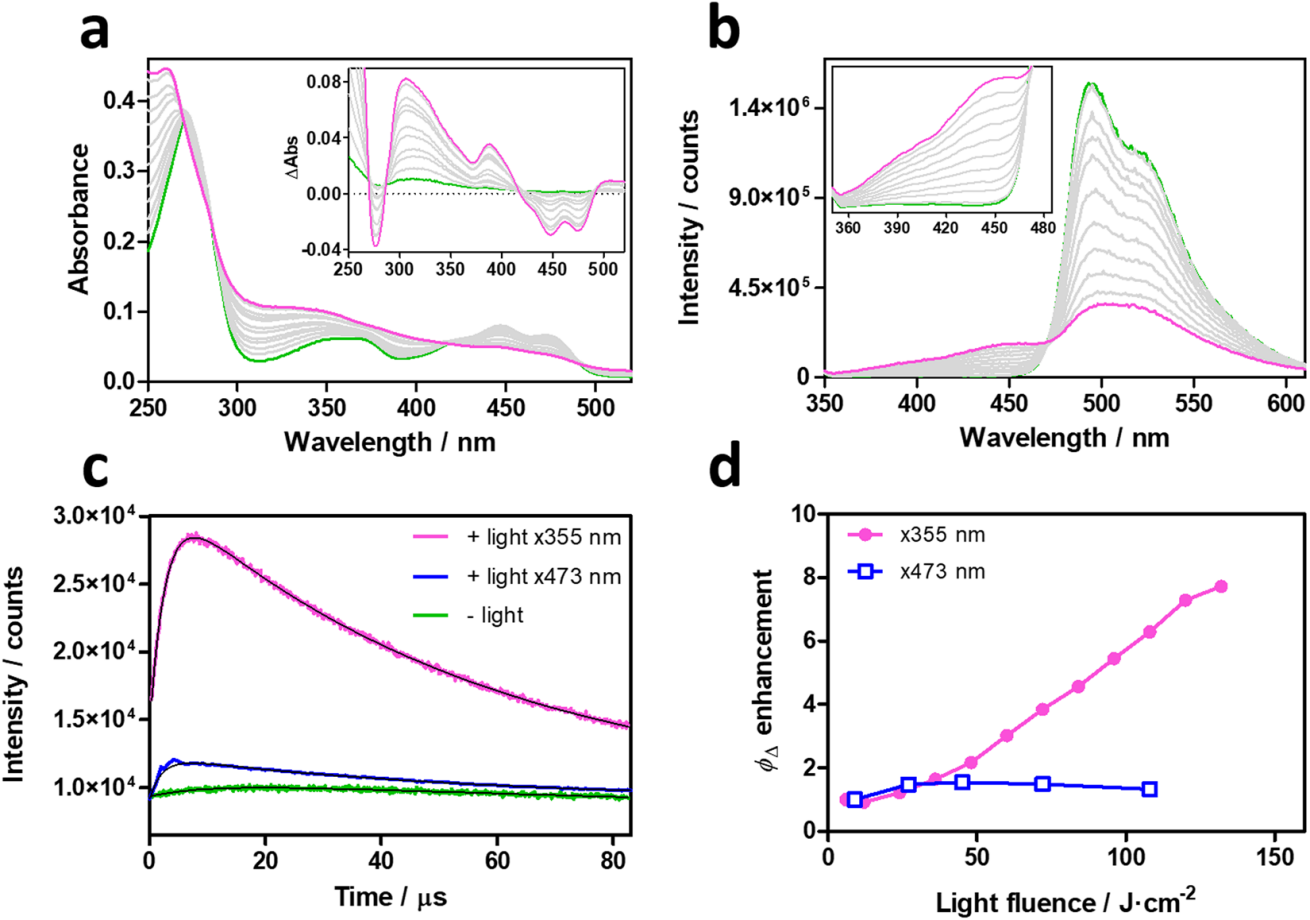
Spectroscopic characterization of photoconverted miniSOG. Evolution of miniSOG’s absorbance (**a**) and fluorescence (**b**) upon laser irradiation at 355 nm. Insets show the difference absorption spectra before and after irradiation, and a zoomed-in image of the new fluorescence bands. (**c**) Time-resolved NIR ^1^O_2_ phosphorescence decays of native (green) and photoconverted miniSOG excited at 473 nm (blue) or 355 nm (magenta). (**d**) Observed *Φ*_Δ_ enhancement at 473 nm (blue) and 355 nm (magenta).

To get further insights into the photoconversion, we performed additional photophysical investigations. Besides the already-known shortening of τ_T_ and increase in *Φ*_∆_, exposure of miniSOG samples to light induces photobleaching of the FMN chromophore and appearance of new absorption and fluorescence bands (Fig. 4a,b). The leaching out of FMN from miniSOG was routinely checked and could be safely ruled out. The quantum yield and rate constant of pathway III could be estimated (Table 1, Supplementary Fig. S1). Noteworthy, the *Φ*_∆_ value increases when the photoconverted miniSOG is excited at 355 nm, but remains essentially constant when probed at 473 nm (Fig. 4c,d).

**Figure 5:**
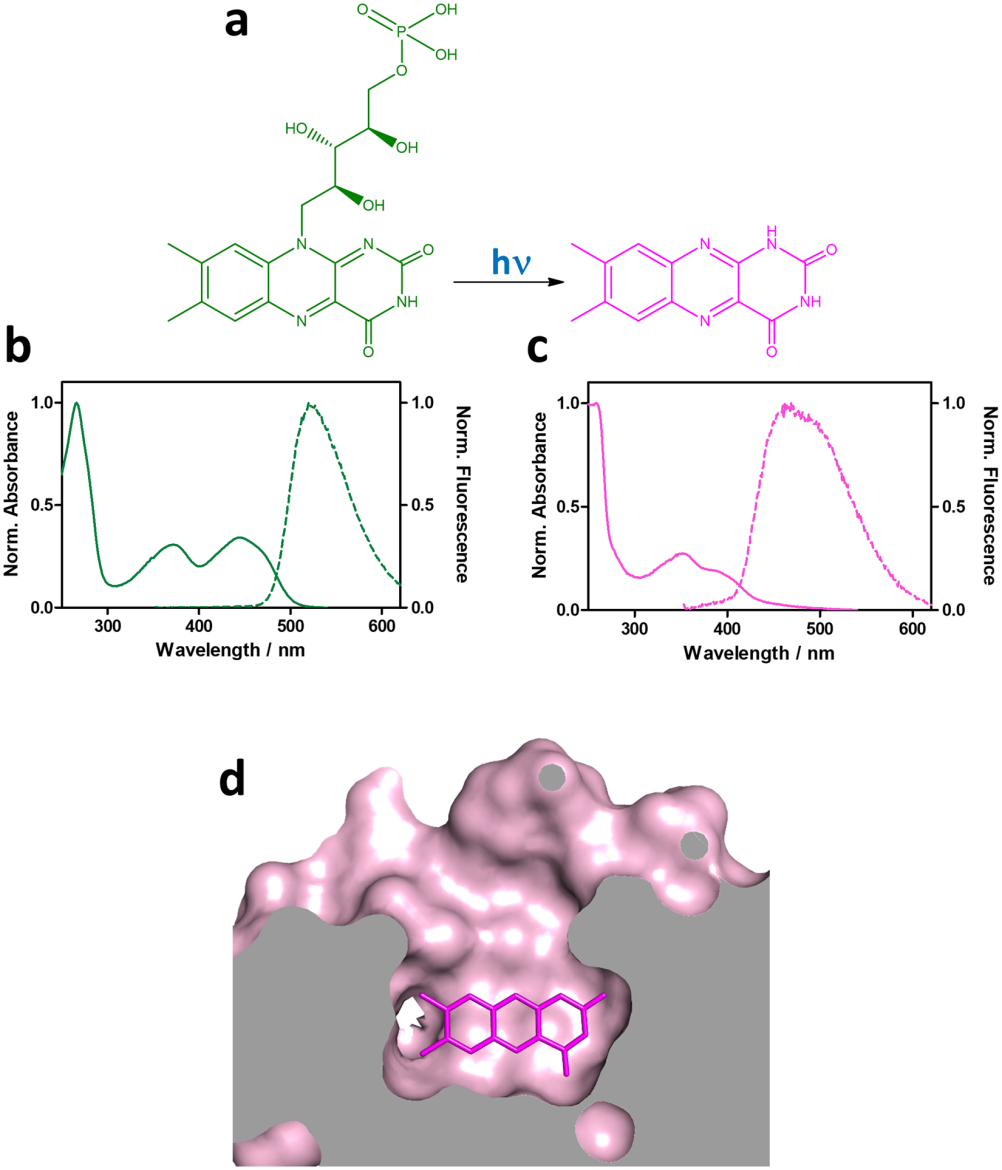
FMN is photoconverted to lumichrome. (**a**) Photoconversion of FMN (green) to LC (magenta). Normalized absorption and fluorescence spectra of (**b**) FMN and (**c**) LC. (**d**) Topology of blue-light irradiated miniSOG showing the increased access to the alloxazine ring.

Phototransformation of FMN to lumichrome (LC) is consistent with all of the above observations: (1) LC is a photodegradation product of flavins in aqueous solutions^24^; (2) the observed mass loss upon irradiation matches the molar mass difference between FMN (456.3 Da) and LC (242.2 Da); (3) LC absorbs and fluoresces at shorter wavelengths than FMN, (Fig. 5); (4) LC lacks the phosphoribityl tail of FMN, which facilitates the access of molecular oxygen to the isoalloxazine ring, resulting in the increase of ${k}_{{{\rm{O}}}_{2}}$ and the decrease of τ_T_; (5) LC is a worse electron acceptor than FMN, hence protein quenching is less favored. The Δ_r_*G*° value for quenching of ^3^riboflavin* by tryptophan is −86.5 kJ·mol^−1^ (riboflavin is analogous to FMN except for the phosphate group) while is more positive for ^3^LC*, −67.2 kJ·mol^−1^ ^25^; (6) finally, LC is also an excellent ^1^O_2_ photosensitizer^25–27^, hence the combination of a higher ${k}_{{{\rm{O}}}_{2}}$ and a lower *k*_P_ yield a higher *Φ*_∆_ value (Eq. 1) when excited at 355 nm but not at 473 nm, where LC barely absorbs.

### Consequences of blue-light irradiation of miniSOG on its amino acid residues (Pathway IV)

**Figure 6:**
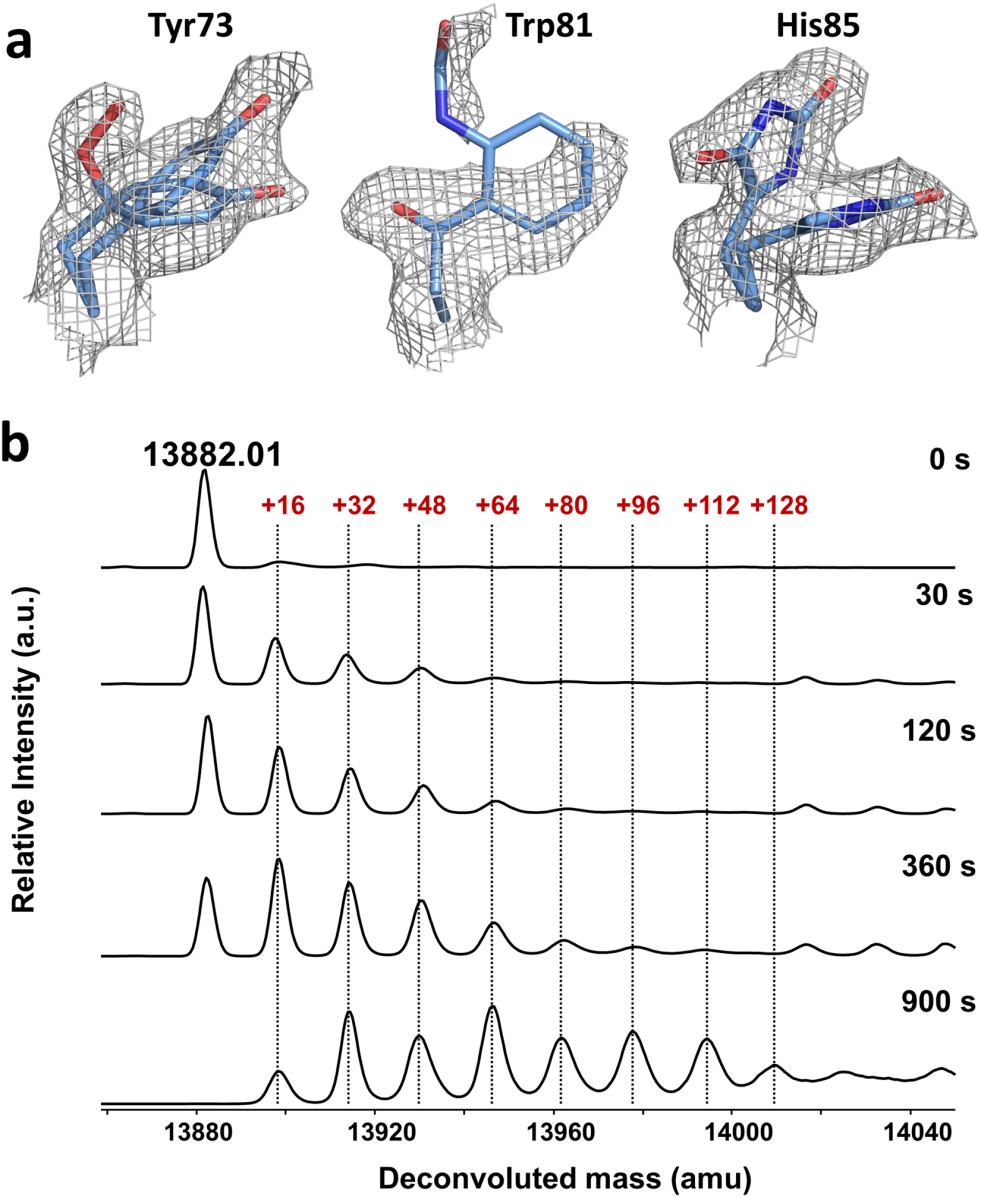
Identification of protein residue oxidation in blue-light irradiated miniSOG. (**a**) 2*F*_obs_ − *F*_calc_ electron density maps contoured at a 1.0, 0.6 and 1.0 σ level superimposed on the refined model of residues Tyr73, Trp81, and His85, respectively. (**b**) Deconvoluted ESI-TOF mass spectra of miniSOG progressively irradiated with blue-light. The peak at 13882.0 Da corresponds to the native protein.

We investigated if our structural data could also support a decrease in *k*_*P*_. Indeed, the 2*F*_obs_ − *F*_calc_ electron density map of blue-light irradiated miniSOG reveals the unambiguous oxidation of three surface residues during irradiation (Fig. 6a, Supplementary Fig. S4). Tyr73 has been partially converted to a γ-peroxotyrosine. The loss of electron density on Trp81 is compatible with the formation of *N*-formylkynurenine (NFK), a well-known tryptophan oxidation product^28,29^. Finally, His85 can be modeled by either a singly, or a doubly oxidized histidine, namely 2-oxo-histidine and 2,4-dioxo-histidine. Mass spectrometry analysis of blue-light irradiated miniSOG samples reveals sequential additions of +16 mass units to the native protein mass of 13882.0 Da, consistent with increasing oxidation steps of the protein (Fig. 6b). All three structural modifications account for six of the eight additional oxygen atoms evidenced in the mass spectrometry analysis. The two non-assigned additions could correspond to oxidation of Tyr30, Met89 or Tyr98, although we did not observe unambiguous oxidation of these residues. Oxidation of Tyr73, His85 and Trp81 eliminates potential quenchers of ^3^miniSOG*, thereby decreasing the value of *k*_p_. According to Eq. 1, this should contribute to an increase in *Φ*_∆_. However, since protein oxidation (pathway IV) occurs simultaneously to FMN → LC transformation, which also increases *Φ*_∆_, it is not possible to ascertain the individual contribution of both effects.

Finally, oxidation of tryptophan into NFK could contribute to the increased *Φ*_∆_ value observed at 355 nm since NFK is a potent singlet oxygen photosensitizer (*Φ*_∆_ = 0.17)^30^. However, the W81F mutant shows a doubled *Φ*_∆_ (=0.33) already before photolysis on account of its lower *k*_p_ value (Eq. 1), indicating that the potential benefits of producing NFK as secondary photosensitizer are of minor value as compared to the effect of eliminating a protein quencher.

## Conclusion

We have performed an extensive structural characterization of miniSOG in the dark and its photoproduct formed in the presence of molecular oxygen, which led us to explain in structural terms the details of its complex photophysical behavior. miniSOG is initially moderately efficient towards ^1^O_2_ generation because of a combination of limited oxygen accessibility and ^3^FMN quenching by electron-rich side chains. Prolonged irradiation to blue light leads to several structural alterations of miniSOG, which include photodegradation of FMN into LC and oxidation of the quenching side chains. All this results in an increase of *Φ*_∆_ when photoconverted miniSOG is excited at the wavelengths where the formed LC absorbs. Formation of LC liberates the access of molecular oxygen to the alloxazine ring and reduces protein quenching of the triplet state, while oxidized electron-rich side chains cannot quench the triplet state of the chromophore. The competition between oxygen quenching and protein quenching of flavin triplet state seems to be a general feature of flavin-binding proteins^31^, hence our results will be useful to guide the evolution of such a protein towards retaining or gaining a specific function. Finally, our results explain the apparent discrepancy between the poor level of singlet oxygen generation by miniSOG, which had been consistently measured at low light fluences, and its efficiency in CLEM experiments, in which the singlet oxygen generation capability of miniSOG is exploited over its whole lifetime.

## Methods

### Chemical compounds

Riboflavin-5′-monophosphate sodium salt hydrate (FMN) (Chemochroma), Lumichrome (Santa Cruz Biotechnology), tris(hydroxymethyl)aminomethane (Merck), sodium chloride (Panreac Applichem), imidazole (Merck), L-arabinose (Sigma Aldrich) and ampicillin (Sigma Aldrich) were used as received. Phosphate-buffered saline (PBS) or deuterated dPBS solutions were prepared by dissolving the required amount of a PBS tablet (Sigma Aldrich) in milliQ water or deuterium oxide (Sigma-Aldrich).

### Expression and purification

Genes coding for a C-terminal 6xHis-tagged recombinant miniSOG and miniSOG W81F were inserted in a pBad expression vector and over-expressed in *Escherichia coli* CodonPlus (DE3) RIL Cells (Stratagene) or in TOP10 cells (Invitrogen). Bacterial cells were grown in LB broth medium containing 1 mM Ampicillin. At an OD_600_ of approximately 0.6, expression of recombinant protein was induced by the addition of L-arabinose and cells were grown for an additional 24 h at 25 °C. Cells were pelleted by centrifugation (4000 g, 4 °C, 30 min), re-suspended in *buffer A* (20 mM Tris-Hcl pH 8.0, 500 mM NaCl), complemented with complete protease inhibitors-EDTA (Roche) and disrupted using a micro-fluidizer. The soluble fraction was recovered by centrifugation (40,000 g, 4 °C, 30 min), and loaded on a 1 mL Ni-NTA superflow column (Qiagen) pre-equilibrated with *buffer A*. The His-tagged protein was eluted with 150 mM imidazole in *buffer A*. Fractions containing purified proteins were pooled and concentrated to a volume of 0.5 mL using Centricon devices (Amicon 10 kDa cut-off) and loaded onto a size-exclusion chromatography column (Hiload Superdex75 10/300, GE Healthcare) for the final step of the purification procedure. The column was equilibrated with 20 mM Tris-HCl pH 8.0 and the pooled peak fractions were concentrated to 4 mg·mL^−1^. Protein expression and purification was always performed in the dark or under red light. The purity of the protein solutions was confirmed by SDS-PAGE. The final concentration was determined by UV-vis absorption spectroscopy using a molar absorption coefficient of 14 mM^−1^·cm^−1^ at 448 nm.

### Spectroscopic measurements

All spectroscopic measurements were performed using quartz cuvettes (Hellma) under magnetic stirring and at room temperature. Absorption spectra were recorded on a double beam Cary 6000i spectrophotometer (Varian). Fluorescence spectra were measured on Fluoromax-4 spectrofluorometer (Horiba). Time-resolved near-infrared (NIR) phosphorescence signals at 1275 nm were measured using a customized PicoQuant Fluotime 200 lifetime system. Briefly, an AO-Z-473 solid state AOM Q-switched laser (Changchun New Industries Optoelectronics Technology Co., China) was used for excitation at 473 nm, working at 1.0 kHz repetition rate at 473 nm. The average power that reached the sample was conveniently modulated by neutral density filters. For excitation at 355 nm, the frequency-tripled output of a diode-pumped pulsed Nd:YAG laser (FTSS355-Q, Crystal Laser, Berlin, Germany) was used, working at 1 kHz repetition (0.5 mW, or 5 mW, 1 ns pulse width). An uncoated SKG-5 filter (CVI Laser Corporation, Albuquerque, U.S.A.) was placed at the exit port of the laser to remove any NIR component. The luminescence exiting from the sample was filtered by a 1100 nm long-pass filter (Edmund Optics, York, U.K.) and a narrow bandpass filter at 1275 nm (bk-1270-70-B, bk Interfernzoptik, Germany) to remove any scattered laser radiation and isolate the ^1^O_2_ emission. A TE-cooled near-IR sensitive photo multiplier tube assembly (H9170-45, Hamamatsu Photonics Hamamatsu City, Japan) in combination with a multichannel scaler (NanoHarp 250, PicoQuant Gmbh, Germany) was used as photon-counting detector. The time-resolved ^1^O_2_ emission decays were analyzed by fitting Eq. 2 ^32^ to the data using GraphPad Prism 5.

2 $${S}_{(t)}={S}_{(0)}\frac{{\tau }_{{\rm{\Delta }}}}{{\tau }_{{\rm{\Delta }}}-{\tau }_{{\rm{T}}}}({e}^{\frac{-t}{{\tau }_{{\rm{\Delta }}}}}-{e}^{\frac{-t}{{{\rm{\tau }}}_{{\rm{T}}}}})\,$$

*τ*_T_ and *τ*_∆_ are the lifetimes of the photosensitizer triplet state and of ^1^O_2_, respectively, and S_(0)_ is a quantity proportional to *Φ*_∆_. *Φ*_∆_ values were determined by comparing the S_(0)_ values of optically-matched solutions of the corresponding flavoprotein and FMN at 473 nm (Eq. 3)^32^.

3 $${\varphi }_{{\rm{\Delta }},protein}=\frac{{S}_{(0)protein}}{{S}_{(0)FMN}}{\varphi }_{{\rm{\Delta }},FMN}\,$$

FMN was taken as reference photosensitizer with *Φ*_∆_ = 0.51 in PBS^11^ and 0.57 in dPBS^33^.

Transient absorption spectra were monitored by nanosecond laser flash photolysis using a Q-switched Nd-YAG laser (Surelite I-10, Continuum) operating at the 3^rd^ harmonic. The luminescence exiting from the sample was filtered by a 610 nm long-pass filter (CVI Laser Corporation, NM, USA). Changes in the sample absorbance were detected at 715 nm using a Hamamatsu R928 photomultiplier to monitor the intensity variations of an analysis beam produced by a 75 W short arc Xe lamp (USHIO) and spectral discrimination was obtained using a PTI 101 monochromator. The signal was fed to a Lecroy Wavesurfer 454 oscilloscope for digitizing and averaging (typically 10 shots) and finally transferred to a PC for data storage and analysis. The system was controlled using the in-house-developed LKS software (LabView, National Instruments).

### Determination of *k*_Phot_

The rate constant for the photoproduct formation has been determined measuring the progressive loss of miniSOG in solution as a function of the absorbed light dose at 473 nm using Eq. 4. The slope of the resulting plot yielded the photobleaching quantum yield *Φ*_Phot_, from which the rate constant for photobleaching was calculated as:

4 $${k}_{Phot}=\frac{{{\rm{\Phi }}}_{Phot}}{{{\tau }_{T}}^{Air}}$$

### X-ray crystallography

#### Crystallization procedures

miniSOG was concentrated to 4 mg·mL^−1^. The crystallization condition consisted of 100 mM Tris-HCl pH 8.0, 20 mM MgCl_2_, 28% PEG 4000, 0 or 15 mM CoCl_2_ at 20 °C. Crystals appeared and grew to final size after 1–2 days.

#### Data collection and processing

X-ray data were collected on beamlines ID23-1^34^ and ID29^35^ of the ESRF and were indexed, integrated, merged and scaled using the *XDS* software package^36^. Molecular replacement was carried out using the model structure of LOV2 (PDB ID: 4eep) with the program *Phaser MR*^37^. Structure refinement was performed using *Refmac5*^38^ and manual improvement of the model with *Coot*^39^. The native structure of miniSOG was used as a starting model for model building of bleached-miniSOG. Data collection and refinement statistics are presented in Supplementary Table S1. Structure analysis and representation were performed with *Pymol*^40^.

### Preparation of photobleached miniSOG samples

#### miniSOG crystals

A single miniSOG crystal was soaked in a cryoprotectant solution containing 20% of glycerol then harvested with a nylon loop. The crystal was exposed to 440 nm laser (10 W·cm^−2^) on the ID29S-Cryobench setup^41^ at room temperature using a HC1 humidity control device^42^. Spectra were recorded at a 1 Hz rate. After 30 min of total exposure, the crystal was flashcooled in liquid nitrogen.

#### miniSOG solutions

Fresh miniSOG or miniSOG W81F solutions in air-saturated deuterated PBS were illuminated at 355 nm (~5 mW·cm^−2^) or 473 nm (~15 mW·cm^−2^) for elapsed irradiation times. Absorption and fluorescence spectra as well as time-resolved ^1^O_2_ phosphorescence decays were recorded at different time intervals of cumulative irradiation.

### Liquid chromatography-mass spectrometry (LC-MS)

Liquid Chromatography Electrospray Ionization Mass Spectrometry (LC/ESI-MS) was carried out on a 6210 LC/ESI-TOF mass spectrometer interfaced with a binary HPLC pump system (Agilent Technologies). The mass spectrometer was calibrated in the positive ion mode with ESI-L (low concentration tuning mix, Agilent Technologies) before each series of measurements, the calibration providing mass accuracy <1 ppm in the 100–3200 *m/z* range. All solvents used were HPLC grade: water and acetonitrile (LC-MS Chromasolv, Sigma-Aldrich); formic acid was from Acros Organics (puriss., p.a.).

Data acquisition was carried out in the positive ion mode with spectra in the profile mode and mass spectra were recorded in the 130–2000 *m/z* range. The mass spectrometer was operated with the following experimental settings: ESI source temperature was set at 325 °C; nitrogen was used as drying gas (5 L/min) and as nebulizer gas (30 psi); the capillary needle voltage was set at 3500 V. Fragmentor value was of 250 V and skimmer of 65 V. The instrument was operated in the 2 GHz (extended dynamic range) mode and spectra acquisition rate was of 1 spectrum/s.

Before analysis, miniSOG samples were diluted to a final concentration of 10 µM in acetonitrile/water/formic acid (50:50:0.1, *v*/*v*/*v*) and infused directly in the mass spectrometer by a syringe pump at a flow rate of 10 μl/min. A blank run was carried out infusing only protein buffer diluted at the same ratio as the protein sample in the same solvent system.

The MS data were acquired and processed with the MassHunter workstation software (Data acquisition v.B.04.00, Qualitative analysis with Bioconfirm v.B.07.00, Agilent Technologies).

### Protein Data Bank accession codes

The structures of non-irradiated and blue-light irradiated miniSOG have been deposited in the Protein Data Bank under entry codes 6GPU and 6GPV, respectively.

## Supplementary information

Supplementary Information
