# Supplementary material for: Tailing miniSOG: structural bases of the complex photophysics of a flavin-binding singlet oxygen photosensitizing protein
Source: Sci Rep. 2019 Feb 20;9:2428. doi: 10.1038/s41598-019-38955-3 (PMC6382843; doi:10.1038/s41598-019-38955-3)
Supplement: Supplementary file 1 — Supplementary Information [file 41598_2019_38955_MOESM1_ESM.docx]

**Supplementary information to “Tailing miniSOG: structural bases of the complex photophysics of a flavin-binding singlet oxygen photosensitizing protein”**

Joaquim Torra^1^, Céline Lafaye^2^, Luca Signor^2^, Sylvain Aumonier^3^, Cristina Flors^4,5^, Xiaokun Shu^6,7^, Santi Nonell^1^*, Guillaume Gotthard^3^*, Antoine Royant^2,3^*

^1^Institut Químic de Sarrià, Universitat Ramon Llull, Via Augusta 390, Barcelona 08017, Spain

^2^Univ. Grenoble Alpes, CNRS, CEA, IBS (Institut de Biologie Structurale), F-38000 Grenoble, France

^3^European Synchrotron Radiation Facility, F-38043 Grenoble, France

^4^Madrid Institute for Advanced Studies in Nanoscience (IMDEA Nanoscience), Ciudad Universitaria de Cantoblanco, C/ Faraday 9, 28049 Madrid, Spain

^5^Nanobiotechnology Unit Associated to the National Center for Biotechnology (CNB-CSIC-IMDEA), Ciudad Universitaria de Cantoblanco, 28049 Madrid, Spain

^6^Department of Pharmaceutical Chemistry, University of California-San Francisco, San Francisco, California 94158-9001, United States

^7^Cardiovascular Research Institute, University of California-San Francisco, San Francisco, California 94158-9001, United States

* Corresponding authors e-mail: [santi.nonell@iqs.url.edu](mailto:santi.nonell@iqs.url.edu), [guillaume.gotthard@esrf.fr](mailto:guillaume.gotthard@esrf.fr) and [antoine.royant@ibs.fr](mailto:antoine.royant@ibs.fr)

**Contents**

Supplementary Results Page 2

Supplementary Tables and Figures Page 3

Supplementary References Page 8

**Supplementary results**

**Analysis of the 1.17 Å structure of miniSOG**

The structure of miniSOG was solved with the molecular replacement method using a previously determined structure of the LOV2 domain of *A. thaliana* phototropin 2 (PDB 4eep).^1^ The structures are very close from each other (r.m.s. deviation on C*α* atoms of 0.39 Å) and we have identified the two mutations C40/426G (miniSOG/phototropin 2 numbering) and F84/470L to affect significantly the FMN-binding cavity. C40G is the photoreaction-hindering mutation, and the removal of a side chain at position 40, added to the smaller size of the side chain at position 84, induce the displacement of three strands towards the isoalloxazine ring, providing the chromophore with a smaller binding pocket, thus with a more rigid environment. A chloride ion is located next to the ribityl chain positioned halfway through the tunnel. The presence of magnesium and chloride is favored by the composition of the crystallization mother liquor. We note that the chloride ion can be a good mimic of molecular oxygen,^2,3^ which suggests that this location may serve as a transient affinity site for oxygen when diffusing from the solvent to the chromophore via the tunnel. Finally, the chromophore is stabilized by the protein through hydrogen bonds on one half of the isoalloxazine ring, and van der Waals interactions on the other half.

**Mass spectrometry analysis of irradiated miniSOG**

In order to identify the precise location of the cleavage, we performed ESI TOF mass spectrometry analysis on protein samples that had been exposed to increasing blue-light doses, acquiring the spectral data in the low mass range region (*m/z* < 500). We could observe the progressive disappearance of the FMN signal, corresponding to the [M+H]^+^ ion at *m*/*z* = 457.1 in favor of a [M+H]^+^ ion at *m*/*z* = 243.1, which corroborates the interpretation that lumichrome (LC, 242.2 Da) is the main photoproduct.

**Supplementary tables and figures**

**Table S1: Data collection and refinement statistics**

| **Data collection** | **miniSOG** | **Blue-light irradiated miniSOG** |
| --- | --- | --- |
| PDB code | 6GPU | 6GPV |
| ESRF Beamline | ESRF ID23-1 | ESRF ID29 |
| Wavelength (Å) | 0.9763 | 0.9763 |
| Space group | P 4_3_ 2_1_ 2 | |
| Cell dimensions  *a, b, c* (Å) | 40.02, 40.02, 134.29 | 40.49 40.49 133.74 |
| Resolution range (Å) | 80 – 1.17 (1.20 – 1.17) | 80 – 2.0 (2.1 – 2.0) |
| Wilson B-factor (Å^2^) | 16.235 | 31.821 |
| Unique reflections | 37913 (2747) | 8116 (1064) |
| Multiplicity | 8.2 (8.0) | 8.2 (8.7) |
| Completeness (%) | 99.8 (99.9) | 99.8 (100.0) |
| Mean I/sigma(I) | 22.1 (2.0) | 12.7 (2.5) |
| *R_meas_* | 0.062 (1.205) | 0.176 (1.06) |
| CC_1/2_ | 1.0 (0.704) | 0.997 (0.735) |
| Resolution (Å) | 38.35 - 1.17 (1.20 – 1.17) | 38.76 – 2.0 (2.05 – 2.0) |
| *R_work_* / *R_free_* | 0.132 (0.234) / 0.164 (0.266) | 0.180 (0.254) / 0.240 (0.278) |
| N. of atoms | 1253 | 1172 |
| Average atomic *B*-factor (Å^2^) | 15.170 | 34.467 |
| R.m.s deviations |  |  |
| Bond lengths (Å) | 0.012 | 0.010 |
| Bond angles (°) | 1.70 | 1.55 |

**Figure S1.** Photobleaching of miniSOG as a function of the absorbed light dose at 473 nm. The intercept yields the photobleaching quantum yield (*Φ*_Phot_ = 2.3 x 10^-4^), which, combined with a triplet lifetime of 38.6 µs, yields (equation S3) the rate constant for photobleaching (*k*_Phot_ = 6.0 s^-1^).

| **Table S2. ^1^O_2_ signal fitting results** | | | |
| --- | --- | --- | --- |
|  | ***τ*_T_ / μs** | ***τ*_∆_ / μs** | ***Φ*_∆_** |
| miniSOG W81F | 265 | 45 | 0.33 |
| FMN | 3.2 | 62 | 0.57^4^ |

**Figure S2.** Transient absorption decays of miniSOG W81F in (**a**) argon-saturated and (**b**) air-saturated dPBS. Fitting lines are in red. (λ_ex_ = 355 nm, λ_obs_ = 715 nm). (**c**) Time-resolved ^1^O_2_ NIR phosphorescence of optically matched solutions of miniSOG W81F (blue) and FMN (red) in air-saturated dPBS-solutions. Fitting lines are in black. Y axis is in log scale.

**
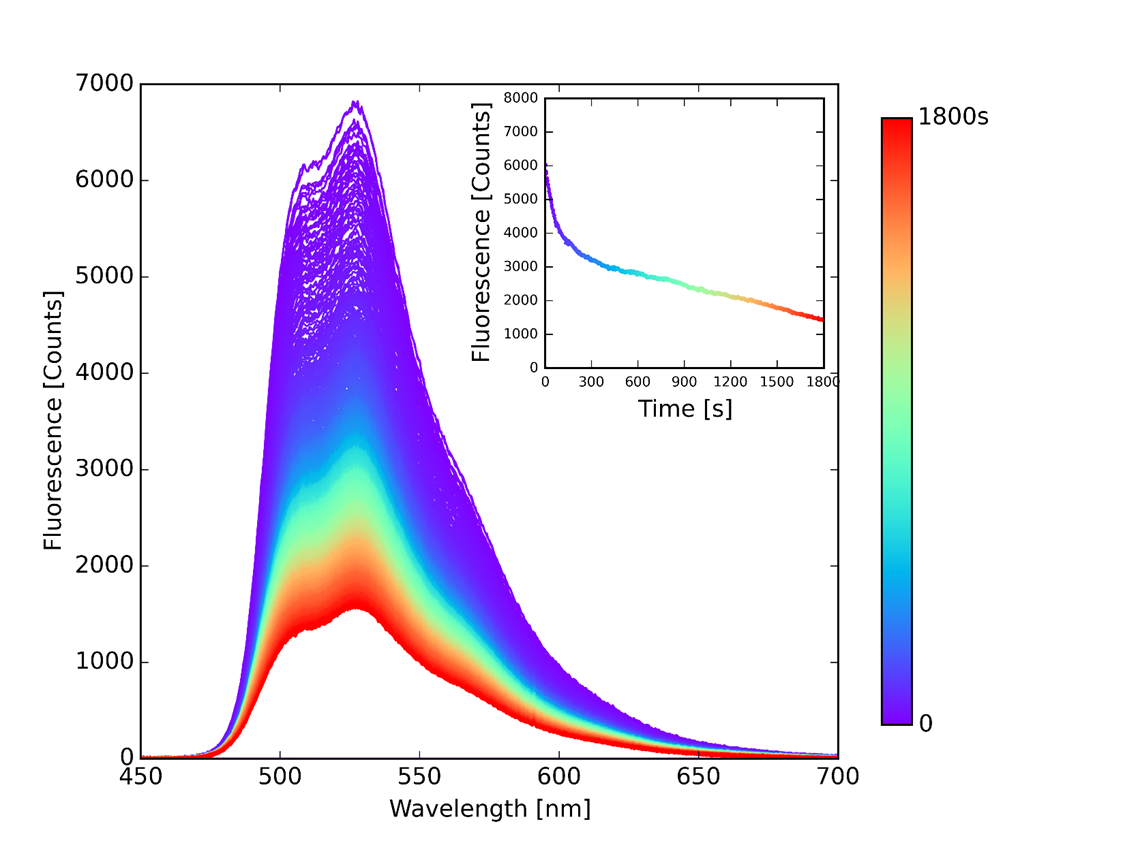
**

**Figure S3:** Time evolution of the fluorescence emission spectrum of a miniSOG crystal under sequential blue light irradiation of a 440 nm laser. Inset shows the decay of the integrated fluorescence signal.

**
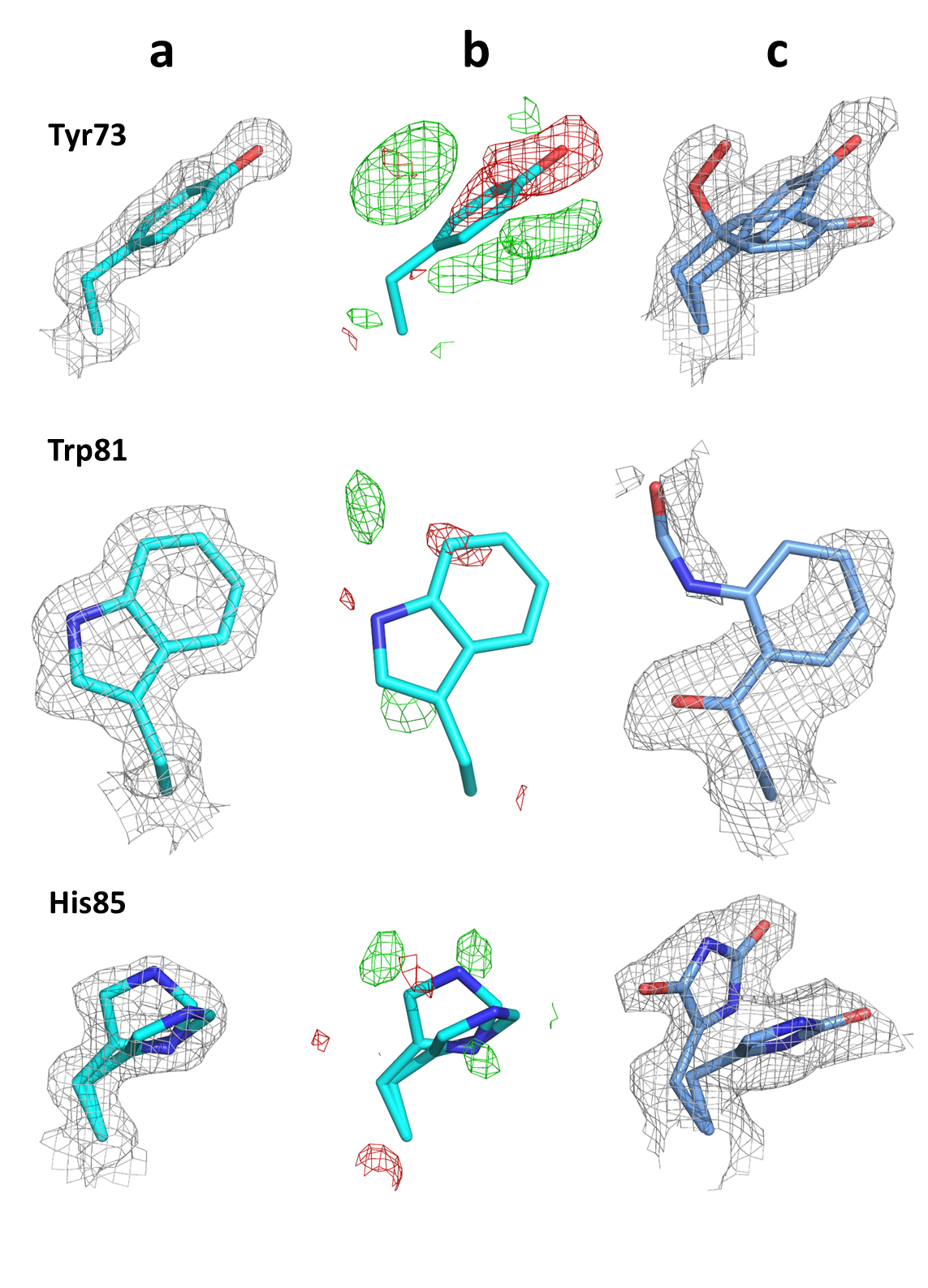
Figure S4:** **a.** 2*F*_obs_ – *F_calc_* electron density map contoured at a 1.0 σ level in the 1.17 Å native structure of miniSOG superimposed on the protein model for Tyr73, Trp81 and His85 (from top to bottom). **b.** Difference Fourier electron density map contoured at a 2.5, 2.0 and 2.5 σ level calculated between data sets recorded on a non-irradiated part, and a blue-light irradiated part of a crystal for Tyr73, Trp81 and His85, respectively. **c.** 2*F*_obs_ – *F_calc_* electron density map contoured at a 1.0, 0.6 and 1.0 σ level in the 2.0 Å structure of blue-light irradiated miniSOG superimposed on the protein model for Tyr73, Trp81 and His85, respectively. Tyr73 is partially modelled as a γ-peroxotyrosine, Trp81 is modelled as a N-formylkynurenine and His85 is modelled partially as a singly oxidized histidine (2-oxo-histidine) and as a doubly oxidized histidine (2,4 dioxo-histidine).

**Supplementary references**

1. Christie, J. M. *et al.* Structural tuning of the fluorescent protein iLOV for improved photostability. *J. Biol. Chem.* **287,** 22295–22304 (2012).

2. Roeser, D., Schmidt, B., Preusser-Kunze, A. & Rudolph, M. G. Probing the oxygen-binding site of the human formylglycine-generating enzyme using halide ions. *Acta Crystallogr. D Biol. Crystallogr.* **63,** 621–627 (2007).

3. Colloc’h, N. *et al.* Oxygen pressurized X-ray crystallography: probing the dioxygen binding site in cofactorless urate oxidase and implications for its catalytic mechanism. *Biophys. J.* **95,** 2415–22 (2008).

4. Rodríguez-Pulido, A. *et al.* Assessing the potential of photosensitizing flavoproteins as tags for correlative microscopy. *Chem. Commun.* **52,** 8405–8408 (2016).
